# Spironolactone reduces biochemical markers of bone turnover in postmenopausal women with primary aldosteronism

**DOI:** 10.1007/s12020-020-02348-8

**Published:** 2020-06-27

**Authors:** Christian Adolf, Leah T. Braun, Carmina T. Fuss, Stefanie Hahner, Heike Künzel, Laura Handgriff, Lisa Sturm, Daniel A. Heinrich, Holger Schneider, Martin Bidlingmaier, Martin Reincke

**Affiliations:** 1 Medizinische Klinik und Poliklinik IV, Klinikum der Universität, LMU München, Ziemssenstraße 1, 80336 Munich, Germany; 2 Medizinische Klinik und Poliklinik I, Schwerpunkt Endokrinologie und Diabetologie, Universitätsklinikum Würzburg, 97080 Würzburg, Germany

**Keywords:** Aldosterone, Osteocalcin, Osteoporosis, Hyperparathyroidism, Cortisol

## Abstract

**Context:**

Primary aldosteronism (PA) is the most frequent form of endocrine hypertension. Besides its deleterious impact on cardiovascular target organ damage, PA is considered to cause osteoporosis.

**Patients and methods:**

We assessed bone turnover in a subset of 36 postmenopausal women with PA. 18 patients had unilateral PA and were treated by adrenalectomy, whereas 18 patients had bilateral PA and received mineralocorticoid receptor antagonist (MRA) therapy respectively. 18 age- and BMI-matched females served as controls. To estimate bone remodeling, we measured the bone turnover markers intact procollagen 1 N-terminal propeptide, bone alkaline phosphatase, osteocalcin and tartrate resistant acid phosphatase 5b in plasma by chemiluminescent immunoassays at time of diagnosis and one year after initiation of treatment.

**Study design:**

Observational longitudinal cohort study.

**Setting:**

Tertiary care hospital.

**Results:**

Compared with controls, patients with PA had mildly elevated osteocalcin at baseline (*p* = 0.013), while the other bone markers were comparable between both groups. There were no differences between the unilateral and the bilateral PA subgroup. One year after initiation of MRA treatment with spironolactone bone resorption and bone formation markers had significantly decreased in patients with bilateral PA. In contrast, patients adrenalectomized because of unilateral PA showed no significant change of bone turnover markers.

**Conclusion:**

This study shows that aldosterone excess in postmenopausal women with PA is not associated with a relevant increase of bone turnover markers at baseline. However, we observed a significant decrease of bone markers in patients treated with spironolactone, but not in patients treated by adrenalectomy.

## Introduction

Primary aldosteronism (PA) represents the most common cause of endocrine arterial hypertension and affects about 5–10% of hypertensives [1, 2]. Moreover, PA has frequently been shown to induce target organ damage independent of blood pressure levels and is associated with metabolic changes including type II diabetes mellitus and osteoporosis [3–6].

From rodent studies we know that aldosterone/salt treatment generates an increase in calciuresis resulting in a decline in plasma calcium concentration. These changes are linked to the development of secondary hyperparathyroidism resulting in a loss of bone mineral density, which could be attenuated by administration of spironolactone in the rodent model [7, 8]. Several studies provide evidence that aldosterone excess also plays a role in human bone health, reporting higher risk of bone and especially vertebral fractures in patients suffering from PA [9–11]. Interestingly, despite higher rates of bone fractures in patients with PA, data on bone mineral density show conflicting results with only small changes on bone mineral density [9, 10, 12]. These data lead to the hypothesis that PA could have higher impact on bone microarchitecture instead of bone mass, which could be illustrated by Kim and colleagues using trabecular bone score [12].

Another possibility to assess microarchitectural alterations affecting bone quality is the measurement of bone turnover markers [13]. To date, to our knowledge, two studies have analyzed bone turnover markers in patients with PA, with conflicting results. In this context, Ceccoli et al. did not detect significant differences for bone turnover markers in 116 patients with PA at time of diagnosis compared with 110 patients with essential hypertension. Similarly, follow-up of 40 patients either adrenalectomized for PA (*n* = 16; ADX) or treated with mineralocorticoid receptor antagonists (*n* = 24; MRA) yielded no differences [14]. Contrary, Loh et al. reported higher levels of bone formation marker intact procollagen I N-terminal propeptide (PINP) and resorption marker carboxy-terminal collagen crosslinks (CTX-1) in 18 patients with PA compared with 17 patients with essential hypertension. Furthermore, they found a significant decrease in PINP and CTX-1 following specific treatment by unilateral ADX (*n* = 3) or MRA (*n* = 12) [15]. These conflicting results may have been influenced by both insufficient matching for sex, BMI, and age at baseline and furthermore by not taking into account sex or gonadal status as well as treatment strategy, either ADX or MRA, for PA [14, 15].

Based on the limited data and the relevance of the issue our aim was to analyze the impact of aldosterone excess on bone remodeling assessed by bone turnover markers in a well-defined collective of 36 postmenopausal women with both unilateral and bilateral PA.

## Methods

The study population consisted of 36 consecutively enrolled postmenopausal women with PA (18 with unilateral and 18 with bilateral PA), who were recruited through the Munich center of the German Conn’s Registry. The focus was laid on postmenopausal females to study a collective with endogenously increased bone turnover which might be more sensitive to aldosterone effects than premenopausal females or males. 18 age- and BMI-matched female controls were recruited in parallel and included in our analysis after exclusion of Cushing syndrome and other endocrinopathies. At baseline, patients with PA and controls were receiving antihypertensive treatment in most cases. In each subgroup three patients were on diuretics, with five patients taking hydrochlorothiazide and one patient torasemide. Furthermore, two patients with PA were on a stable regimen of hormone replacement therapy for menopausal symptoms, which was continued until the end of the study. Similarly, at follow-up three patients with PA were on diuretics. All patients gave written informed consent, and the ethics committee of the University of Munich approved the protocols.

All patients underwent a standardized procedure including biochemical screening, physical examination and anthropometric measurements. 24-hour urinary collection was conducted at each visit to estimate daily sodium intake. To screen for subclinical hypercortisolism cortisol after 1 mg low dose overnight dexamethasone suppression test (LDDST), as well as measurement of 24-h urinary cortisol excretion and sampling of late-night salivary cortisol were performed.

Bone remodeling was assessed by three bone formation markers: PINP, osteocalcin, and bone alkaline phosphatase (BAP) as well as the bone resorption marker tartrate resistant acid phosphatase 5b (TrAP). Samples were analyzed at our Endocrine Laboratory on the iSYS automated analyzer (IDS-iSYS, Boldon, UK) by well-validated assays [16–19]. To minimize preanalytical confounding an N-MID assay was used to determine osteocalcin concentration.

Diagnosis of PA was made in accordance with the Endocrine Society Practice Guidelines [20]. In brief, after elevated aldosterone to renin ration (ARR; cut-off 12.0 ng/U, sitting position) in initial screening the diagnosis of PA was confirmed by an abnormal confirmatory test (e.g., salt loading test, captopril challenge test or both). Antihypertensive medication was discontinued before testing, if possible. Otherwise it was replaced by alpha 1-adrenergic receptor (doxazosin) or calcium-channel blockers (verapamil) in most cases. The subtype diagnosis of PA was based on adrenal vein sampling as published earlier [21]. Patients with unilateral disease were only included in the analysis, if they underwent ADX, otherwise they were excluded from the analysis. All patients with bilateral disease were treated with MRA, using spironolactone at a dose of 25–50 mg/d.

Patients with PA were re-evaluated 12 months after treatment in an identical fashion.

### Statistical analysis

All values are expressed as median, 25th and 75th percentile if not mentioned otherwise. Body mass index (BMI) was calculated as weight in kilograms divided by the square of the height in meters. Data between groups were compared using Mann–Whitney *U* test. Within-group changes from baseline to follow-up were calculated by Wilcoxon signed-rank test. Spearman’s Rank Order was used to perform bivariate correlation analysis.

Stepwise multiple regression analysis was performed for multivariate analysis. Two-tailed probability values of <5% were considered to be statistically significant. Statistical analysis was performed using standard statistical software (SPSS 25, IBM, Chicago, IL).

## Results

In total, data of 54 postmenopausal women, 36 with PA and 18 controls, was analyzed. None of the participants had a history of non-traumatic bone fracture, received specific osteoanabolic or antiresorptive treatment or had been diagnosed with osteoporosis at baseline. However, 8 patients with PA and 2 controls received vitamin D supplementation (*p* = 0.322), with patients with PA having higher levels of 25-hydroxyvitamin D compared with controls (28.9 ng/ml vs 19.0 ng/ml; *p* = 0.021). Most other baseline parameters, like BMI, age as well as features of calcium metabolism were comparable between the groups. Likewise, 24-h urinary sodium and cortisol excretion as well as late-night salivary cortisol did not differ between the groups. However, cortisol after LDDST was significantly higher in patients with PA (*p* = 0.006).

At baseline, patients with PA had mildly elevated bone formation marker osteocalcin compared with controls (*p* = 0.023), while all other bone turnover markers were comparable between the groups (Table 1).

**Table 1:** Baseline and follow-up characteristics of patients with primary aldosteronism and controls

Patient characteristics	Patients with PA at baseline (*n* = 36)	Patients with PA at follow-up (*n* = 36)	*p*	Controls (*n* = 18)	*p*
Age [years]	59 [53; 64]	–	–	54 [44; 60]	0.057
BMI [kg/m^2^]	26.0 [23.2; 30.0]	26.2 [23.1; 30.1]	0.946	27.0 [25.9; 34.8]	0.099
Serum sodium [mmol/l]	141 [139; 143]	139 [137; 141]	**0.002**	141 [139; 143]	0.897
Serum potassium [mmol/l]	3.8 [3.4; 4.3]	4.4 [4.1; 4.6]	**<0.001**	4.4 [4.0; 4.6]	**0.001**
Serum creatinine [mg/dl]	0.8 [0.7; 0.9]	0.9 [0.7; 1.0]	**<0.001**	0.8 [0.7; 0.9]	0.963
Serum calcium [mmol/l]	2.4 [2.4; 2.5]	2.5 [2.4; 2.6]	**0.001**	2.5 [2.4; 2.5]	0.255
Serum phosphate [mg/dl]	3.3 [2.9; 3.7]	3.5 [3.0; 3.7]	0.271	3.2 [3.0; 3.3]	0.434
25-hydroxyvitamin D [ng/ml]	28.9 [17.2; 37.6]	27.2 [18.5; 34.2]	0.417	19.0 [9.8; 25.5]	**0.021**
Parathyroid hormone [mg/dl]	53.5 [41.0; 66.8]	47.6 [36.5; 59.5]	0.106	66.3 [38.2; 75.3]	0.370
HbA1c [%]	5.5 [5.0; 5.9]	5.6 [5.2; 5.8]	**0.049**	5.8 [5.3; 6.0]	0.242
Diabetes mellitus [%]	17	–	–	17	1.000
BAP [µg/l]	17.0 [13.9; 21.7]	16.9 [11.8; 22.3]	0.068	17.8 [13.3; 20.9]	1.000
Osteocalcin [ng/ml]	20.6 [14.4; 32.0]	14.6 [9.1; 23.2]	**0.013**	12.4 [10.8; 18.2]	**0.023**
PINP [ng/ml]	55.1 [42.8; 80.5]	41.4 [29.0; 57.7]	**0.005**	50.1 [34.7; 75.8]	0.419
TrAP [U/l]	2.3 [1.8; 4.4]	2.2 [1.7; 3.4]	0.071	2.1 [1.5; 2.8]	0.189
LDDST [µg/dl]	1.5 [1.2; 2.1]^a^	–	–	1.1 [0.9; 1.6]	**0.006**
UFC [µg/d]	107 [60; 151]^a^	–	–	105 [77; 201]	0.419
Late-night salivary cortisol [ng/ml]	1.4 [1.0; 2.1]^a^	–	–	1.6 [1.1; 2.6]	0.622
24-h urinary calcium [mmol/d]	4.6 [3.6; 6.6]	2.8 [1.7; 4.1]	**<0.001**	–	–
24-h urinary sodium [mmol/d]	152 [105; 208]	140 [98; 181]	0.460	163 [123; 213]	0.557
Estimated salt intake [g/d]	8.9 [6.1; 12.2]	8.2 [5.7; 10.6]	0.460	9.5 [7.2; 12.5]	0.557

Data are given as median, 25th and 75th percentile in square brackets. Significance is marked in bold. Comparisons between baseline values of both groups were performed by Mann–Whitney *U* test, comparisons to baseline values by Wilcoxon signed-rank test. – data not available/not calculated

*BAP* bone alkaline phosphatase, *HbA1c* glycated hemoglobin, *LDDST* cortisol after 1 mg low dose overnight dexamethasone suppression test, *PINP* intact procollagen I N-terminal propeptide, *TrAP* tartrate resistant acid phosphatase 5b, *UFC*: 24-h urinary cortisol excretion

^a^Data set of 33 patients with complete data

In PA patients BAP (*r* = 0.53; *p* = 0.001) and osteocalcin (*r* = 0.45; *p* = 0.006) strongly correlated with increasing age, whereas there was no significant correlation of neither bone formation nor bone resorption markers with parameters of hypercortisolism, including DSST, late-night salivary cortisol and 24-h urinary cortisol excretion in univariate analysis (Supplementary Table 1). Moreover, 24-h urinary sodium excretion was strongly correlated with 24-h urinary calcium excretion (*r* = 0.53; *p* = 0.001) but not with bone turnover markers.

Patients with unilateral PA showed significantly higher aldosterone levels (*p* = 0.002) at baseline than bilateral patients. Otherwise, both cohorts were comparable for most anthropometric and biochemical data as well as bone turnover parameters (Table 2). One year after initiation of treatment, either by ADX or by MRA, serum potassium was normalized (ADX: *p* = 0.001; MRA: *p* = 0.001) and 24-h urinary calcium excretion (ADX: *p* = 0.002; MRA: *p* = 0.028) as well as parathyroid hormone levels were reduced with the latter not reaching statistical significance (ADX: *p* = 0.248; MRA: *p* = 0.267), while levels of 25-hydroxyvitamin D were unchanged in both subgroups (Table 2).

**Table 2:** Baseline and follow-up characteristics of patients with primary aldosteronism according to subtype

Patient characteristics	Patients with unilateral PA (*n* = 18)		*p*	Patients with bilateral PA (*n* = 18)		*p*
Time of assessment	Baseline	After ADX		Baseline	After MRA	
Age [years]	60 [53; 64]	–	–	59 [54; 65]	–	–
BMI [kg/m^2^]	26.7 [23.7; 28.9]	26.1 [23.6; 29.8]	0.917	26.0 [22.9; 31.6]	26.2 [23.1; 30.5]	0.937
Serum sodium [mmol/l]	142 [140; 143]	140 [138; 140]	**0.003**	140 [139; 143]	139 [137; 142]	0.100
Serum potassium [mmol/l]	3.7 [3.2; 4.0]	4.3 [4.1; 4.5]	**0.001**	3.8 [3.8; 4.3]	4.4 [4.2; 4.7]	**0.001**
Serum creatinine [mg/dl]	0.8 [0.6; 0.9]	0.9 [0.7; 0.9]	**0.002**	0.8 [0.7; 0.9]	0.9 [0.8; 1.2]	**0.001**
Serum calcium [mmol/l]	2.4 [2.3; 2.5]	2.4 [2.4; 2.5]	0.070	2.5 [2.4; 2.5]	2.5 [2.4; 2.6]	**0.009**
Serum phosphate [mg/dl]	3.3 [3.0; 3.5]	3.4 [3.0; 3.7]	0.312	3.5 [2.9; 3.9]	3.6 [3.3; 4.0]	0.585
25-hydroxyvitamin D [ng/ml]	23.5 [13.1; 39.7]	25.2 [18.1; 38.1]	0.632	31.2 [21.4; 37.5]	29.7 [26.2; 33.9]	0.463
Parathyroid hormone [mg/dl]	56.1 [45.6; 71.0]	48.6 [38.0; 67.2]	0.248	47.4 [39.1; 57.8]	47.4 [32.6; 55.4]	0.267
HbA1c [%]	5.5 [5.2; 6.0]	5.6 [5.3; 5.8]	0.167	5.5 [5.0; 5.8]	5.6 [5.2; 6.2]	0.124
Diabetes mellitus [%]	17	–	–	17	–	–
BAP [µg/l]	17.2 [15.4; 21.3]	17.6 [14.4; 23.1]	0.420	15.7 [12.8; 24.7]	14.8 [9.8; 19.3]	**0.004**
Osteocalcin [ng/ml]	19.4 [14.8; 32.3]	17.7 [10.6; 28.3]	0.286	21.3 [11.7; 32.0]	11.8 [8.5; 21.2]	**0.018**
PINP [ng/ml]	54.4 [45.3; 73.1]	49.7 [32.0; 70.7]	0.215	57.5 [31.3; 81.5]	36.6 [23.9; 48.1]	**0.007**
TrAP [U/l]	2.1 [1.4; 4.4]	2.5 [1.9; 4.1]	0.828	2.4 [2.0; 4.5]	2.1 [1.6; 2.7]	**0.028**
LDDST [µg/dl]	1.8 [1.3; 3.3]^a^	–	–	1.5 [1.2; 2.1]	–	–
UFC [µg/d]	96 [45; 140]^a^	–	–	111 [73; 166]	–	–
Late-night salivary cortisol [ng/ml]	1.3 [0.8; 2.1]^a^	–	–	1.6 [1.1; 2.1]	–	–
24-h urinary calcium [mmol/d]	4.8 [3.9; 6.6]	2.9 [1.8; 3.6]	**0.002**	4.0 [3.3; 7.8]	2.8 [1.3; 4.7]	**0.028**
24-h urinary sodium [mmol/d]	172 [103; 237]	123 [85; 180]	**0.014**	129 [103; 169]	146 [121; 186]	0.122
Estimated salt intake [g/d]	10.0 [6.0; 13.8]	7.2 [4.9; 10.5]	**0.014**	7.5 [6.0; 9.9]	8.5 [7.0; 10.8]	0.122

Data are given as median, 25th and 75th percentile in square brackets. Significance is marked in bold. Comparisons between baseline values of both groups were performed by Mann–Whitney *U* test, comparisons to baseline values by Wilcoxon signed-rank test. – data not available/not calculated

*BAP* bone alkaline phosphatase, *HbA1c* glycated hemoglobin, *LDDST* cortisol after 1 mg low dose overnight dexamethasone suppression test, *PINP* intact procollagen I N-terminal propeptide, *TrAP* tartrate resistant acid phosphatase 5b, *UFC* 24-h urinary cortisol excretion

^a^Data set of 15 patients with complete data

**Fig. 1:**
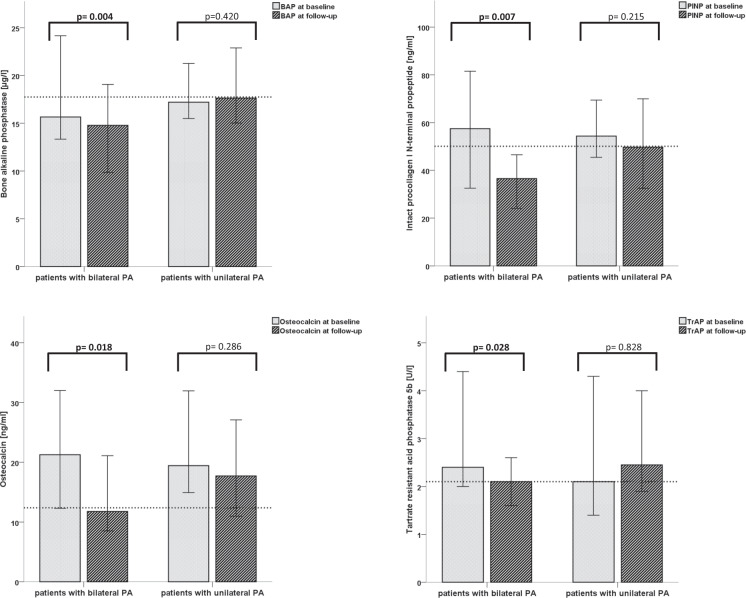
Median of bone turnover markers before and after specific treatment for primary aldosteronism according to subtype diagnosis. Significance is marked in bold. Median of controls is illustrated in dashed lines

Interestingly in patients with bilateral disease bone turnover markers osteocalcin (*p* = 0.018), PINP (*p* = 0.007), BAP (*p* = 0.004) as well as TrAP (*p* = 0.028) decreased significantly (Table 2), in contrast to patients treated with ADX in whom bone formation and bone resorption makers were unaltered (Fig. 1).

## Discussion

The main findings of our study are twofold: at baseline, postmenopausal female patients with PA only show a mild increase in bone turnover markers compared with matched controls. One year after initiation of treatment, MRA therapy in bilateral PA was associated with decreased bone turnover markers, a phenotype that was not seen following ADX in case of unilateral PA. We conclude from these data, that MRA therapy using spironolactone has a potential bone protective effect.

Apart from its deleterious effects on cardiovascular target organs, PA is acknowledged as a relevant cause of secondary osteoporosis [4, 22, 23]. Several mechanisms have been postulated to explain higher rates of osteoporosis in PA. Beside indirect effects of PA through increased calciuresis and secondary hyperparathyroidism, also direct effects on bone metabolism have been proposed as mineralocorticoid receptors have been identified in human bone cells [14, 24, 25]. Furthermore, in genome-wide association studies a strong connection between genes belonging to aldosterone signaling and bone strength was found [26]. Cortisol cosecretion, which is a typical feature of PA, represents another mediator, which could affect bone health in PA a fortiori as its effects of the latter on glucose metabolism and cardiac structure have already been described [27–29]. Last but not least high dietary salt intake has been reported to promote bone loss in postmenopausal women, which is indeed controversially discussed [30–33].

Our data show that bone metabolism in PA is characterized by slightly increased bone formation parameter osteocalcin at baseline, while bone resorption marker TrAP was unaltered.

High dietary sodium intake, estimated by 24-h urinary sodium excretion, was correlated with 24-h urinary calcium excretion, which has been frequently observed [34]. In this context, assuming a negative calcium balance, there have been several studies reporting an inverse relationship for salt intake and bone mineral density [33–35]. However, observational data remain heterogeneous and interventional data to date are still missing [36]. Besides, we could not detect any direct correlation between urinary sodium excretion and bone turnover markers neither at baseline nor at follow-up. In this context, high dietary salt intake in patients with PA could impact bone health by promoting calciuresis but this needs to be further validated in interventional trials.

Concerning the effects of hypercortisolism on bone health, several studies revealed decreased bone formation parameters in patients with Cushing syndrome [22]. For osteocalcin there has even a negative correlation with cortisol at late-night and after DSST been described, whereas data on bone resorption parameters are heterogeneous [37, 38]. Data on bone turnover markers in patients suffering from mild autonomous cortisol secretion are inconsistent, most likely due to sample size and selection criteria [39–41]. We therefore investigated the impact of cortisol cosecretion, which is a frequent finding in PA, on bone turnover parameters [27]. In the PA cohort data on LDDST, 24-h urinary cortisol excretion and late-night salivary cortisol was available in 33 of 36 patients. There was no correlation for any of those parameters with markers of bone turnover or 24-h urinary calcium excretion in univariate analysis. In addition, the combination of pathological LDDST, 24-h urinary cortisol excretion or late-night cortisol did not show significant differences in bone turnover markers. In summary we could not find a (relevant) impact of cortisol cosecretion on bone turnover markers in our small cohort of PA patients, with the latter apparently being a limiting factor.

Patients with bilateral PA undergoing MRA treatment had a significant decrease of BAP, osteocalcin and PINP as well as the bone resorption marker TrAP, which is in accordance with findings from Loh et al. [15]. In contrast, patients with unilateral disease who underwent ADX demonstrated no significant changes in bone turnover markers. This rather unexpected result could bring different findings from Loh and Ceccoli together. This is based on the fact that Ceccoli, who studied a cohort of PA patients with a high proportion of patients with unilateral PA (40%) could not detect differences in bone turnover markers, whereas Loh, who found a significant decrease in bone turnover markers after treatment, analyzed a cohort of 15 patients including only a small proportion of patients (*n* = 3; 20%) undergoing adrenalectomy. Therefore, we speculate that the high proportion of patients receiving spironolactone could have counterbalanced small changes in bone turnover markers in unilateral PA patients by Loh when analyzing the PA cohort without considering different treatment modalities.

Based on the findings that in postmenopausal women higher bone turnover markers are supposed to indicate rapid bone loss and bone markers being higher in women after fractures those findings could reflect an improvement of bone quality and probably also in bone mass in patients with PA undergoing treatment with MRA but not with ADX [42, 43]. Spironolactone itself has been frequently reported to improve bone health in different cohorts of patients [15, 44, 45]. One major point of the bone protective effect of spironolactone could be its antimineralocorticoid effect, enabling enhanced tubular reabsorption of calcium resulting in higher serum calcium levels and a decrease in PTH, which would be in line with effects of thiazide diuretics which have been shown to be beneficial for bone health in the setting of hypertension [8, 46, 47]. Furthermore, spironolactone reduces urinary magnesium and potassium excretion, which might have additional bone protective effects in patients with chronic heart failure and MRA therapy [45, 48–52]. In PA, long-term spironolactone treatment could have similar favorable effects on bone health, a theoretical advantage compared with unilateral adrenalectomy [3].

Apart from its antimineralocorticoid effects spironolactone has been reported to attenuate the increase in bone turnover in context with GnRH therapy, why it has been speculated that it could have effects on estrogen and progestogen receptors comparable with selective estrogen receptor modulators resulting in increased bone mass [44, 53]. Another point could be the fact that the MR has been reported to be expressed on osteoblasts and osteocytes [24]. Although, to date, the function remains to be elucidated, it has been shown that treatment by eplerenone reduced in part glucocorticoid-induced osteopenia and therefore it has been speculated that MRA treatment could affect not only aldosterone-mediated but also glucocorticoid-mediated effects on bone health [54].

In summary, our data from a well-characterized cohort of postmenopausal women with PA show a presumably non-relevant effect of aldosterone excess on bone turnover markers. This illustrates that bone turnover at time of diagnosis of PA is not the major factor for changes in bone microarchitecture and vertebral fractures in PA. The strong correlation between 24-h urinary sodium and calcium excretion could indicate a direct effect of high salt diet on bone health in PA. Specific treatment, either by ADX or by MRA, was followed by a highly significant decrease in 24-h urinary calcium excretion, which was shown to be associated with improved bone health [14]. Furthermore, treatment with spironolactone was associated with a highly significant decrease in bone turnover markers. In context with heterogenous and in part conflicting results from other studies concerning the effect of specific treatment for PA on the risk of bone fractures, our study adds further evidence that MRA treatment could be effective for the prevention of osteoporosis in patients with PA [11, 14]. However, further prospective trials are necessary to improve early diagnosis and treatment strategies for patients with PA, who are beyond doubt at higher risk for bone fracture.

Our study results are limited by the exploratory nature of a well-characterized but rather small cohort of PA patients. We acknowledge that observational studies as ours deal with uncertainties by itself. Therefore, we cannot fully exclude that our results could have been confounded by distortion effects. Furthermore, the assessment of study data was performed using post-hoc analysis and for instance bone mineral density could not be assessed. This study also has several strengths including the standardized collection of all data and biomaterial within the context of the German Conn’s and German Cushing Registries, the homogeneously characterized study population, and the subtyping of all patients by adrenal vein sampling.

## Supplementary material

Supplementary Table 1
