# Correction: Spironolactone reduces biochemical markers of bone turnover in postmenopausal women with primary aldosteronism

**DOI:** 10.1007/s12020-021-02905-9

**Published:** 2021-10-23

**Authors:** Christian Adolf, Leah T. Braun, Carmina T. Fuss, Stefanie Hahner, Heike Künzel, Laura Handgriff, Lisa Sturm, Daniel A. Heinrich, Holger Schneider, Martin Bidlingmaier, Martin Reincke

**Affiliations:** 1 Medizinische Klinik und Poliklinik IV, Klinikum der Universität, LMU München, Ziemssenstraße 1, 80336 Munich, Germany; 2 Medizinische Klinik und Poliklinik I, Schwerpunkt Endokrinologie und Diabetologie, Universitätsklinikum Würzburg, 97080 Würzburg, Germany

Correction to: Endocrine (2020) 69:625–633

10.1007/s12020-020-02348-8

The article Spironolactone reduces biochemical markers of bone turnover in postmenopausal women with primary aldosteronism, was originally published electronically on the publisher’s internet portal on 27 July 2020 without open access. With the author(s)’ decision to opt for Open Choice the copyright of the article changed on 6 October 2021 to © The Author(s) 2021 and the article is forthwith distributed under a Creative Commons Attribution.

**Open Access** This article is licensed under a Creative Commons Attribution 4.0 International License, which permits use, sharing, adaptation, distribution and reproduction in any medium or format, as long as you give appropriate credit to the original author(s) and the source, provide a link to the Creative Commons licence, and indicate if changes were made. The images or other third party material in this article are included in the article’s Creative Commons licence, unless indicated otherwise in a credit line to the material. If material is not included in the article’s Creative Commons licence and your intended use is not permitted by statutory regulation or exceeds the permitted use, you will need to obtain permission directly from the copyright holder. To view a copy of this licence, visit http://creativecommons.org/licenses/by/4.0/.

