# Supplementary material for: Spironolactone reduces biochemical markers of bone turnover in postmenopausal women with primary aldosteronism
Source: Endocrine. 2020 Jun 27;69(3):625–33. doi: 10.1007/s12020-020-02348-8 (PMC8514385; doi:10.1007/s12020-020-02348-8)
Supplement: Supplementary file 1 — Supplementary Table 1 [file 12020_2020_2348_MOESM1_ESM.docx]

**Supplementary Table 1:**

|  | **LDDST** [µg/dl] | **UFC** [µg/d] | **Late-night salivary cortisol** [ng/ml] |
| --- | --- | --- | --- |
| **BAP** [µg/l] | 0.926 | 0.468 | 0.586 |
| **Osteocalcin** [ng/ml] | 0.161 | 0.629 | 0.991 |
| **PINP** [ng/ml] | 0.212 | 0.730 | 0.700 |
| **TrAP** [U/l] | 0.224 | 0.163 | 0.152 |
